# Pathophysiology of chronic limb ischemia

**DOI:** 10.1007/s00772-018-0380-1

**Published:** 2018-04-10

**Authors:** F. Simon, A. Oberhuber, N. Floros, P. Düppers, H. Schelzig, M. Duran

**Affiliations:** 1 Department of Vascular and Endovascular Surgery, Düsseldorf University, Moorenstr. 5, 40225 Düsseldorf, Germany; 2 Network for Fundamental Research in Vascular Medicine (Netzwerk Gefäßmedizinische Grundlagenforschung, NGG), Düsseldorf, Germany

**Keywords:** Pathophysiology, Chronic ischemia, Collaterals, Arteriogenesis, Angiogenesis, Pathophysiologie, Chronische Ischämie, Kollateralen, Arteriogenese, Angiogenese

## Abstract

Chronic ischemia of the lower extremities is an everyday problem in vascular surgery clinics. In Germany, approximately 3% of all hospitalizations are due to peripheral artery disease (PAD), with critical limb ischemia (CLI) in particular showing a rapid increase. The consequences of chronic undersupply range from reduced walking distance to loss of limbs. At the beginning there are stress factors, such as hyperlipidemia (LDL), free radicals, arterial hypertension, infections or subclinical inflammation that interfere with endothelial homeostasis and cause endothelial dysfunction with increased permeability. Cells of the immune system are attracted and migrate into the vascular wall, where they lead to the degradation of matrix components and destabilization of the plaque. By changing the phenotype of smooth muscle cells and macrophages towards osteoclast-like cells, bone-like hardening of the vessel wall takes place. Above a vessel wall thickness of approximately 100 µm, hypoxia-induced factor (HIF-1α) is intensified by the lack of oxygen, which leads to an increase in growth factors, such as vascular endothelial growth factor (VEGF). This promotes angiogenesis, but it is not sufficient to compensate for a stenosed artery. Arteriogenesis refers to the growth of existing collateral vessels. The driving forces are the pressure gradient before and after the stenosis and the shear forces acting on the vessel walls. In the case of progressive stenosis, the compensatory capacities can be overtaxed and a manifest hypoxia in the tissue with regression of the obtained vascular structures and tissue atrophy occurs.

## Zusammenfassung

Die chronisch arterielle Ischämie der unteren Extremitäten stellt ein alltägliches Problem in gefäßchirurgischen Kliniken dar. In Deutschland sind etwa 3 % aller Hospitalisierungen auf eine periphere arterielle Verschlusskrankheit (PAVK) zurückzuführen, wobei vor allem die kritischen Ischämien stark zunehmen. Die Folgen der chronischen Ischämie reichen von eingeschränkter Gehstrecke bis hin zum Verlust der Extremität. Am Anfang stehen Stressfaktoren wie Hyperlipidämie (LDL), freie Radikale, arterielle Hypertonie, Infektionen oder subklinische Entzündungen, die die Endothelhomöostase stören. Es kommt zu einer Dysfunktion des Endothels mit erhöhter Durchlässigkeit. Zellen des Immunsystems werden angelockt und wandern in die Gefäßwand ein, wo sie zum Abbau von Matrixkomponenten und zur Destabilisierung des Plaques führen. Durch Änderung des Phänotyps von glatten Muskelzellen und Makrophagen entstehen osteoklastenähnliche Zellen, die zu Verhärtungen in der Gefäßwand führen. Ab einer Gefäßwandstärke von etwa 100 μm wird durch den Sauerstoffmangel Hypoxie induzierter Faktor (HIF-1α) verstärkt exprimiert, was zu einem Anstieg an Wachstumsfaktoren wie VEGF („vascular endothelial growth factor“) führt. Dadurch wird die Angiogenese als Kapillarenbildner gefördert, reicht aber nicht aus, um eine stenosierte Arterie zu kompensieren. Arteriogenese bezeichnet das Wachstum von bereits vorhandenen Kollateralgefäßen. Die treibenden Kräfte sind der Druckgradient vor und hinter der Stenose sowie die Scherkräfte, die auf die Gefäßwände einwirken. Bei voranschreitender Stenosierung können die Kompensationsfähigkeiten überfordert werden und es kommt zu einer manifesten Hypoxie im Gewebe mit Rückbildung der gewonnenen Gefäßstrukturen/Kollateralkreisläufen und Gewebsatrophie.

## Introduction to the topic

Chronic limb ischemia (CLI) generally occurs in old age and is a common reason for treatment. Due to the slow progression of symptoms, the transition to critical ischemia can appear to “come as a surprise.” It is important to have knowledge and understanding of the pathophysiological processes in limbs damaged by chronic ischemia in order to provide these patients with adequate treatment and prevent or at least delay further damage.

## Introduction

Chronic tissue ischemia in the lower extremities is a common problem encountered in vascular surgical departments. Peripheral arterial occlusive disease (PAOD) affects more than 202 million people worldwide [9]. Approximately 3% of all hospitalizations in Germany are due to PAOD, with a sharp rise being seen in critical ischemia [16]. More than 20% of over 65-year-olds already have PAOD [7], whereby the rate of symptomatic forms in men exceeds 8% [15]. A PAOD is the result of a gradual process that progressively narrows the arterial lumen through which blood flows. This vasoconstriction is generally a sign of atherosclerotic changes in the vessel walls. Typical risk factors include advanced age, nicotine abuse, diabetes mellitus, hypercholesterolemia, and arterial hypertension [18]. The sequelae of chronic ischemia range from reduced pain-free walking distance to loss of the extremity. Therefore, restoring perfusion is the crucial goal and its success depends on numerous factors. The biological adaptation of chronically undersupplied tissue manifests as the formation of a collateral network. This process is more pronounced the longer and, above all, the slower vasoconstriction develops. Angiogenesis, i. e., the growth of small vessels from pre-existing vessels, and arteriogenesis, dilation of the lumen of pre-existing vessels, can lead to the development of a dense collateral network [27]; however, if PAOD progression persists, these biological adaptation mechanisms become overburdened, i. e., collateral supply is no longer sufficient to compensate for tissue hypoperfusion. This results in increasing cell loss, ultimately leading to a local inflammatory reaction. In the course of inflammation, damaged tissue is broken down and replaced by fibrosis. This additional barrier to oxygen diffusion in the tissue further exacerbates ischemia, resulting in a downward spiralling vicious circle. Depending on the degree of stenosis and the biological reaction, different degrees of PAOD severity develop and can affect patients to varying extents. The classification of PAOD according to Fontaine stages ranges from asymptomatic PAOD in stage I and varying degrees of reduced walking distance in stage II, to pain at rest as well as necrosis and gangrene in stages III and IV. As a result, approximately 25,000 patients per year in Germany require a major amputation as a last resort, of which around 70% are diabetics [12]. Other signs can include trophic changes in the affected limb due to impaired macrocirculation and microcirculation. In contrast to venous ulcers, which are primarily located above the medial malleolus, arterial ulcers are predominantly seen at acral sites. Further signs include cold sensation at these sites, as well as metabolic disorders involving skin appendage changes, such as loss of leg hair.

Approximately 25,000 patients per year in Germany require a major amputation

Irrespective of the stage of PAOD, effective treatment of impaired circulation, in conjunction with individual risk factor adjustment, not least due to the risk of ulcer infection, is crucial to the success of treatment for PAOD, which would progressively deteriorate if left untreated.

## Molecular mechanisms of atherosclerosis and atheroma development

A number of factors are expressed in healthy endothelium, such as prostacyclin, nitric oxide (NO), thrombomodulin, and plasminogen activators, all of which have a strongly platelet-repellent, anticoagulant, and fibrinolytic effect. In addition, the endothelium has a high negative charge, explaining why the also negatively charged platelets are repelled from the vessel wall. This makes the luminal side of each vessel a strong barrier to thrombotic factors.

Cell-to-cell contacts are particularly important in the regulation of endothelial permeability. Important complexes for the regulation of these compounds include occludin, adhesion molecules, cadherins, gap junctions, and integrin receptors such as fibronectin [17].

The principal cause of vasoconstriction is atherosclerosis, a chronic process that goes unnoticed for years. The process starts with stress factors such as hyperlipidemia (LDL), free radicals, arterial hypertension, infections, or subclinical inflammation, which impair endothelial homeostasis. This leads to impairment of the stressed endothelium and increased permeability to lipids, which build up in the intima causing further endothelial stress. This mutually reinforcing cycle enables lipids to penetrate deeper into the vascular wall layers and accumulate in the extracellular matrix. Oxidative stress and enzymatic modifications produce oxidized LDL, which triggers strong inflammatory activity [29]. Immune system cells such as monocytes are attracted by these lipid accumulations and migrate into the vascular wall; however, not only the innate but also the acquired immune system contributes to atheroma development by activating T‑cells and antibody production. Cytokines such as interleukin 18 (IL-18) mediate this interaction between different immune cells [1].

Migrated monocytes differentiate into macrophages in the subendothelial tissue, where they can be polarized into different phenotypes depending on the composition of the environment into which they migrate. They express an abundance of receptors in order to absorb the LDL present, whereby the macrophages are converted into foam cells. The release of growth factors and cytokines induces smooth muscle cells to migrate from the media into the intima, where they then form extracellular matrix components, thereby contributing to the formation of the fibrous cap [14]. Many of the foam cells perish in the process themselves and are broken down by other macrophages. Due to the number of these degradation processes, the macrophage endoplasmic reticulum is overburdened leading to further macrophage death. As a result of this now uncontrolled decay, all intracellular components of macrophages, such as lipids, inflammatory cytokines, and metalloproteinases (MMP), are released.

This reaction, which has now become independent, leads to the destruction of matrix components and, thus, to plaque destabilization and ultimately to plaque rupture. This process is additionally driven by smooth muscle cell loss and ischemia-induced angiogenesis [31].

Another process that is already underway in the initial phase of atheroma development takes place simultaneously. As an initial reaction to endothelial stress, intimal thickening occurs with the migration of phenotypically altered vascular smooth muscle cells (VSMC) [32]. These cells produce a milieu rich in proteoglycans and extracellular matrix in their immediate environment, where lipoproteins can accumulate and undergo oxidization to a greater extent. These VSMCs possess an abundance of receptors, such as the low-density lipoprotein receptor-related protein 1 (LRP1) or the lectin-type oxidized LDL receptor 1 (LOX-1), which make increased endocytosis of oxidized lipoproteins possible. At the same time these smooth muscle cells lose transport systems in order to be able to release the enriched lipoprotein once again. These transporters include, e. g., the ATP-binding cassette transporter A1 (ABCA1) and apolipoprotein A1 (ApoA1). As a result of this imbalance, the lipoprotein content in VSMC increases. Thus, the smooth muscle cells are transformed into foam cells and undergo apoptosis. This foam cell decay causes an inflammatory reaction that attracts circulating monocytes via the MCP-1 receptor and further perpetuates inflammation through proinflammatory cytokines such as IL-1α and IL-1β. Thus, a vicious cycle is set in motion that ultimately results in atheromatous changes in the vessel wall [13].

The onset of artherosclerosis is an active process

From this it can be deduced that the risk of fibrous cap rupture has less to do with the degree of vascular stenosis than it does with plaque formation [2]. A thin cap measuring less than 65 μm, a necrotic core occupying more than 30% of the total plaque, hemorrhage, infiltration of inflammatory cells such as lymphocytes and macrophages, as well as the reduction in smooth muscle cells are unfavorable factors that result in rupture and subsequent thrombosis and occlusion of the vessel by destabilizing the plaque and fibrous cap [30]. The structure of the necrotic core is particularly important in this process.

The loss of stabilizing matrix components as well as the build-up of lipids and inflammatory cells causes the core to become mechanically unstable. The apoptotic death of smooth muscle cells as well as macrophages increases this instability and, due to fibrin production, promotes thrombus formation via the release of tissue factor (TF) in the case of rupture [28].

The alteration of the phenotype of smooth muscle cells and macrophages or their polarization also leads to the development of osteoclast-like cells. These cells, in combination with a change in Ca++ and phosphate levels and a decrease in inhibitory cytokines, induce osteogenesis. Calcium-binding protein, osteopontin, and fetuin A are among the messengers that could prevent calcification [21]. Finally, hydroxyapatite crystals are deposited in the tissue. It is important here whether the crystals are deposited in the intima or in the media. Intimal sclerosis is closely linked to atheromatous plaque formation and is assumed to protect the intima from pathological processes in the media, which, due to progressive luminal narrowing, further exacerbates stenosis [21]. Furthermore, calcium deposits in the fibrous cap increase the risk of plaque rupture. This contrasts with medial calcification, which essentially takes place in close relation to smooth muscle cells.

This causes calcium salt deposits, which are subject to constant transformation until bone structures have formed. These deposits contribute more to the reduced elasticity and compliance of the vascular wall itself than they do to the progression of stenosis.

Plaque growth causes the oxygen diffusion distance to increase significantly. Starting at a vessel wall thickness of approximately 100 μm, hypoxia-induced factor (HIF-1α) expression is enhanced due to the lack of oxygen, leading to an increase in growth factors such as vascular endothelial growth factor (VEGF). This promotes angiogenesis to sprout new vessels from pre-existing vessels from the adventitia into the intima. The further the process progresses, the more chaotic growth becomes, leading to immature and unstable vessels that continue to enlarge and destabilize the nucleus through hemorrhage [20].

Other factors that influence arteriosclerotic plaques include stress and shearing forces acting on the vessels. Atherosclerotic changes occur primarily in vascular branches and sections where blood flow is not linear. Low shear stress in particular results in an increased tendency towards inflammation in endothelial cells and leukocytes [22]. Differences in the distribution of inflammatory cells and wall thickening result in characteristic predilection sites for rupture of the various plaques. Plaque rupture caused by physical exertion is more likely to occur at the middle of the atheroma, whereas rupture occurring at rest is more likely to be found in the shoulder region of the plaque. This is explained by the fact that a particularly high number of inflammatory cells settle there, whereas central hardening caused by calcification leads to reduced vessel and plaque elasticity and hence to tearing under stress in particular [3].

If the endothelium breaks down or a plaque layer tears, a chain reaction occurs that culminates in platelet-rich thrombus formation, which further narrows or shifts the vascular lumen. Interaction between a platelet surface glycoprotein (GPIbα) and the von Willebrand factor (vWF) A1 domain occurs in a first step. There are other binding possibilities to leukocytes, macrophages, and *P*-selectin. In a second step, this hitherto reversible adhesion is transformed by the interaction between two platelet collagen receptors (GPIa/IIa and GPVI) into stable thrombus adhesion. This leads, among other things, to the activation of phospholipase C, the mobilization of calcium, as well as the activation of integrins, laminin, and fibronectin, resulting in further platelet activation [10].

## Angiogenesis and arteriogenesis: the basics of collateral formation

The task of blood vessels is to transport oxygen and nutrients to the organs and individual cells, as well as to remove waste products. It is not surprising, therefore, that there is more than one way to ensure perfusion both in healthy and diseased tissue. Vasculogenesis is a process by which vessels with a wholly adequate three-layered wall structure are created without the need for pre-existing vessels. This process is the primary process in the development and growth of new life and plays only a minor role in the adult individual; however, the recruitment of endothelial progenitor cells (EPC) that produce numerous growth factors can result in a milieu of vasculogenesis and angiogenesis in previously avascular tissue [11]. Angiogenesis and arteriogenesis are paramount in wound healing and especially in the chronic hypoperfusion discussed here.

Angiogenesis creates new capillary vessels from existing capillaries, with wound healing or the undersupply of oxygen to tissue representing the driving forces here. To this end, a variety of factors such as fibroblast growth factor (FGF), VEGF, and HIF are released, with HIF stimulating the transcription of VEGF [8]. This causes the proliferation and lateral migration of endothelial cells. The basement membrane and adjacent tissue are eroded by proteases such as metalloproteinases, and the pericytes surrounding the endothelium become detached. The VEGF renders endothelial cells more permeable to plasma proteins, thereby enabling the formation of a rudimentary extracellular matrix in which endothelial cells can in turn be anchored. A pseudopodium extension develops, enabling endothelial cell growth into the surrounding connective tissue. This extension is guided by signal molecules such as ephrin and semaphorin in order to maintain a particular direction [19]. The capillary shoot is the starting point for the accumulation of further cells and, after continuous growth, ultimately forms a lumen. In order to stabilize the new vessel, the endothelial cells return to a resting state, and pericytes, via signal molecules such as platelet derived growth factor B (PDGF-B), transforming growth factor-β (TGF-β), and Notch (transmembrane proteins involved in cell-to-cell communication), adhere to and surround the new vessel. Due to the activation of protease inhibitors such as tissue inhibitors of metalloproteinases (TIMP) and plasminogen activator inhibitor-1 (PAI-1), a basement membrane and new cell contacts are formed [4]; however, angiogenesis is not sufficient as a means of capillary formation to replace a stenosed or occluded artery. This is due to the low flow resistance in arteries that capillaries are unable to compensate with their much higher resistance. A further reason is the long distance between stenosis and hypoperfused tissue, hence the greater relevance attached to arteriogenesis [26].

Hypoxia is a key stimulus of angiogenesis

Arteriogenesis refers to the expansion of existing collateral vessels [24], which are predominantly arched, bypass arterioles within the boundary of various supply regions with low flow and play only a minor role in organ perfusion under healthy conditions. By a process of remodelling, these vessels change their diameter, thereby forming a type of biological bypass that is then extended. The driving forces here include arterial blood pressure, the pressure gradient above and below the stenosis, and the shear stress acting on the vessel walls [25]. The arterioles are expanded by the pressure gradient in a first step, resulting in overexpansion and leakage of plasma proteins. Endothelial cells then enter the cell cycle and smooth muscle cells proliferate and express metalloproteinases (MMPs), which break down the basement membranes and collagen fibers and macerate the tissue. Lymphocytes also enter the tissue and, by killing off surrounding cells, create space for the pending growth of arterioles. Ultimately, the cell mass and total diameter of the individual collaterals can be up to 50 and 20 times, respectively, greater than those of the original arterioles [23]. As a result of endothelium activation, monocytes are attracted by cytokines, such as monocytes of chemoattractant protein 1 (MCP-1), which produce growth factors and proteolytic enzymes that additionally enable smooth muscle cells to migrate and divide. Other factors such as granulocyte macrophage colony-stimulating factor (GM-CSF) prolong the lifespan of monocytes and represent important arteriogenic factors. Since the vessels increase not only in diameter but also in length, yet are located in a restricted environment, the arterioles are subject to substantial distortion, ultimately resulting in the characteristic appearance of collaterals on angiography. All arterioles participate in early arteriogenesis; however, depending on flow, main collaterals quickly emerge that are subjected to maximum stimulation and can then undergo the complete arteriogenic remodelling process. In the case of long-term occlusion, these vessels are able take over a significant proportion of the perfusion of main vessels [26].

## Critical ischemia: the final stage of atherosclerosis

In the case of progressive stenosis, the compensatory capacity of arteriogenesis can be compromised, and manifest tissue hypoxia occurs. Physical activity is restricted due to ischemic pain. This results in reduced stimulation of arteriogenesis and degeneration of the newly formed vascular structures as well as to progressive muscle cell loss, thereby further reducing exercise capacity and setting a downward spiral in motion [5]. Chronic inflammation severely impairs endothelial function. As part of this, free radicals (ROS) attack the vessels’ capacity to relax by intercepting the NO produced by the endothelium for vascular relaxation. Reduced muscle activity leads to lower shear stress in the collaterals, as a result of which the endothelium produces even less NO and superoxide dismutase (SOD), a radical scavenger [6]. Another aspect of the particular conditions seen in chronically undersupplied tissue is that physiological messengers are initially formed in a compensatory capacity but go on to be produced at the cost of residual cell function in the further course. Endothelium-derived relaxing factor (EDRF) is produced by the endothelium to widen vessels and thus increase blood flow. This mechanism to combat ischemia, which is normally only required for short periods, e. g., during muscle exertion in order to keep oxygen debt as low as possible during physical activity, is permanently active in the atherosclerotic patient. This state of permanent activation stimulates cell reduction in other processes such as protein synthesis. This reduction in other cell processes causes intracellular processes that are crucial to survival to gradually diminish, thereby additionally increasing cell stress. Intracellular stress, reduced blood flow, and lowered blood pressure amplitude ultimately result in damage and, in the further course, to atrophy of the vessels and the tissue to be perfused [26].

## Conclusion

- Chronic limb ischemia is a multifactorial phenomenon.
- The onset of atherosclerosis is an active process in which foam cell formation and cell apoptosis play a crucial role.
- Hypoxia is a key stimulus of angiogenesis, whereas shear stress induces arteriogenesis.
- By means of vessel expansion, arteriogenesis is able to take over a significant proportion of the perfusion of major vessels.
- If excessive demands are put on these compensatory mechanisms, tissue atrophy and scar remodelling processes occur.
